# Low doses of X-rays induce prolonged and ATM-independent persistence of γH2AX foci in human gingival mesenchymal stem cells

**DOI:** 10.18632/oncotarget.4739

**Published:** 2015-07-23

**Authors:** Andreyan N. Osipov, Margarita Pustovalova, Anna Grekhova, Petr Eremin, Natalia Vorobyova, Andrey Pulin, Alex Zhavoronkov, Sergey Roumiantsev, Dmitry Y. Klokov, Ilya Eremin

**Affiliations:** ^1^ State Research Center - Burnasyan Federal Medical Biophysical Center of Federal Medical Biological Agency (SRC-FMBC), Moscow, Russia; ^2^ Semenov Institute of Chemical Physics, Russian Academy of Sciences, Moscow, Russia; ^3^ Dmitry Rogachev Federal Research Center of Pediatric Hematology, Oncology and Immunology, Moscow, Russia; ^4^ Moscow Institute of Physics and Technology, Dolgoprudny, Moscow Region, Russia; ^5^ Emanuel Institute for Biochemical Physics, Russian Academy of Sciences, Moscow, Russia; ^6^ Insilico Medicine, Inc, ETC, Johns Hopkins University, Baltimore, Maryland, USA; ^7^ The Biogerontology Research Foundation, BGRF, London, UK; ^8^ N.I. Pirogov Russian National Research Medical University, Moscow, Russia; ^9^ Canadian Nuclear Laboratories, Chalk River, Ontario, Canada

**Keywords:** mesenchymal stem cells, DNA double-strand breaks, DNA repair, X-rays, low doses

## Abstract

Diagnostic imaging delivering low doses of radiation often accompany human mesenchymal stem cells (MSCs)-based therapies. However, effects of low dose radiation on MSCs are poorly characterized. Here we examine patterns of phosphorylated histone H2AX (γH2AX) and phospho-S1981 ATM (pATM) foci formation in human gingiva-derived MSCs exposed to X-rays in time-course and dose-response experiments. Both γH2AX and pATM foci accumulated linearly with dose early after irradiation (5–60 min), with a maximum induction observed at 30–60 min (37 ± 3 and 32 ± 3 foci/cell/Gy for γH2AX and pATM, respectively). The number of γH2AX foci produced by intermediate doses (160 and 250 mGy) significantly decreased (40–60%) between 60 and 240 min post-irradiation, indicating rejoining of DNA double-strand breaks. In contrast, γH2AX foci produced by low doses (20–80 mGy) did not change after 60 min. The number of pATM foci between 60 and 240 min decreased down to control values in a dose-independent manner. Similar kinetics was observed for pATM foci co-localized with γH2AX foci. Collectively, our results suggest differential DNA double-strand break signaling and processing in response to low vs. intermediate doses of X-rays in human MSCs. Furthermore, mechanisms governing the prolonged persistence of γH2AX foci in these cells appear to be ATM-independent.

## INTRODUCTION

Multipotent mesenchymal stromal cells (MSCs) are the most studied and characterized type of stem cells currently used in regenerative medicine [1, 2]. MSCs are described in the literature as substrate-dependent fibroblast-like cells with clonogenic properties and ability to self-renew [3, 4]. These cells express a specific set of surface markers, most of which are common with fibroblasts [5, 6]. MSCs are multipotent and able to differentiate into chondrocyte, osteoblast or adipocyte lineages, also known as the orthodox differentiation. Non-orthodox differentiation in the epithelial, myogenic or neuronal directions have also been shown for MSCs under certain conditions [7, 8].

The main sources of MSCs in the adult are bone marrow, adipose tissue and other connective tissues of the body [9, 10]. Notably, MSCs isolated from different types of tissues can vary greatly in efficiency of differentiation, colony formation and proliferative potential of cells, which determines the effectiveness of MSCs expansion *in vitro* [11, 12]. Other important aspects that can affect selection of the source of MSCs in medical practices are accessibility of the tissue and the level of invasiveness of tissue biopsy sampling [13]. That is why one of the readily available sources of cell material for cell therapy is the oral mucosa (gingiva). Gingival biopsy is minimally traumatic and accompanied by only slight discomfort to the patient, and followed by wound healing without scarring [14]. Besides, gingival-derived MSCs are known to have a number of characteristics that make them very attractive for use in regenerative medicine [15, 16]. Gingival MSCs possess strong immunomodulatory properties [15]. Also, along with the orthodox differentiation, gingival MSCs are arguably able to undergo the non-orthodox differentiation into the neuronal lineage [17, 18] since they originate from the neural crest cells [17]. Furthermore, the oral mucosa pool of MSCs is thought to contain substantially higher number of multipotent stem cells compared with MSCs pools in other tissues [19–21]. For example, up to 90% of the cells in primary cultures of gingival-derived MSCs are characterized by typical stem-cell markers and 40–70% express pluripotency genes Oct4, Sox2 and Nanog [19] and active telomerase [20].

Cell therapy in clinical practice is often accompanied by a variety of X-ray diagnostic procedures during which the tissue is irradiated with low doses (up to 100 mGy). At the same time the stem cells in general, MSCs in particular, are responsible for the ability of tissues to recover following radiation exposure *in vivo*. However, there are substantial knowledge gaps in our understanding of the MSCs response to low dose radiation. Only few and highly controversial reports are available. It was shown that low dose radiation (40 mGy) induces cellular aging and impairs autophagy in human bone marrow MSCs [22]. On the other hand, there is evidence that exposure to X-rays at doses 50 and 75 mGy stimulates proliferation of rat bone marrow MSCs through the activation of MAPK/ERK signaling pathways [23].

Long-term consequences following exposure to radiation are largely defined by cells ability to cope with DNA damage inflicted by irradiation. Although a broad spectrum of DNA lesions, including single- and double-strand breaks (DSBs), base modifications and sugar-phosphate backbone lesions, are typically induced by ionizing radiation, responses to DSBs have a major role in determining the fate of cells [24–28]. Normally, the cell response to ionizing radiation depends on the number of accumulated DSBs and may include processes, such as cell cycle arrest, activation of DNA repair and programs of cell death by autophagy or apoptosis [29, 30]. Previous reports suggest that cells respond differentially to DSBs produced by low vs. high radiation doses [31, 32].

Repair of a DSB is a complex multistep process consisting of initial break recognition followed by a sequence of spatially localized post-translational modifications of various proteins, including histones. This leads to conformational chromatin changes allowing accessibility of the damaged site to repair enzymes. One of the most prominent and studied histone modifications is phosphorylation of the core histone H2AX at serine-139 (called γH2AX) by ataxia telangiectasia mutated (ATM), ataxia telangiectasia and Rad3-related (ATR) kinases and DNA-dependent protein kinase catalytic subunit (DNA-PKcs) that belong to the family of phosphoinositide 3-kinases [33]. Thousands of γH2AX molecules produced in the vicinity of every DSBs form foci that can be visualized using immunofluorescence microscopy [34]. ATM is the main kinase that phosphorylates H2AX around radiation-induced DSBs under physiological conditions [35, 36]. ATR is responsible mainly for the processing of DSBs arising at collapsed replication forks [37, 38], and DNA-PKcs rapidly phosphorylates H2AX in extreme conditions [39]. ATM is also a central kinase phosphorylating key proteins (Chk1, Chk2, Rad17, NBS1, BRCA1, BLM, SMC1, 53BP1, MDC1, p53, MDM2, etc.) involved in various responses to ionizing radiation, such as cell cycle arrest, repair, cell death [40, 41].

The aim of this work was to study patterns of pATM and γH2AX foci formation and loss in cultures of human gingiva-derived MSCs exposed to X-ray radiation. We carried out dose-response and time-course experiments covering low (20, 40 and 80 mGy) and intermediate (160 and 250 mGy) doses and time points of 5, 10, 15, 30, 60, 120 and 240 min. We quantified pATM and γH2AX foci and their co-localization using immunofluorescence microscopy. We show differential γH2AX foci kinetics for low vs. intermediate doses, with the difference appearing to be ATM-independent.

## RESULTS

### Dose-responses for γH2AX and pATM foci

**Figure 1:**
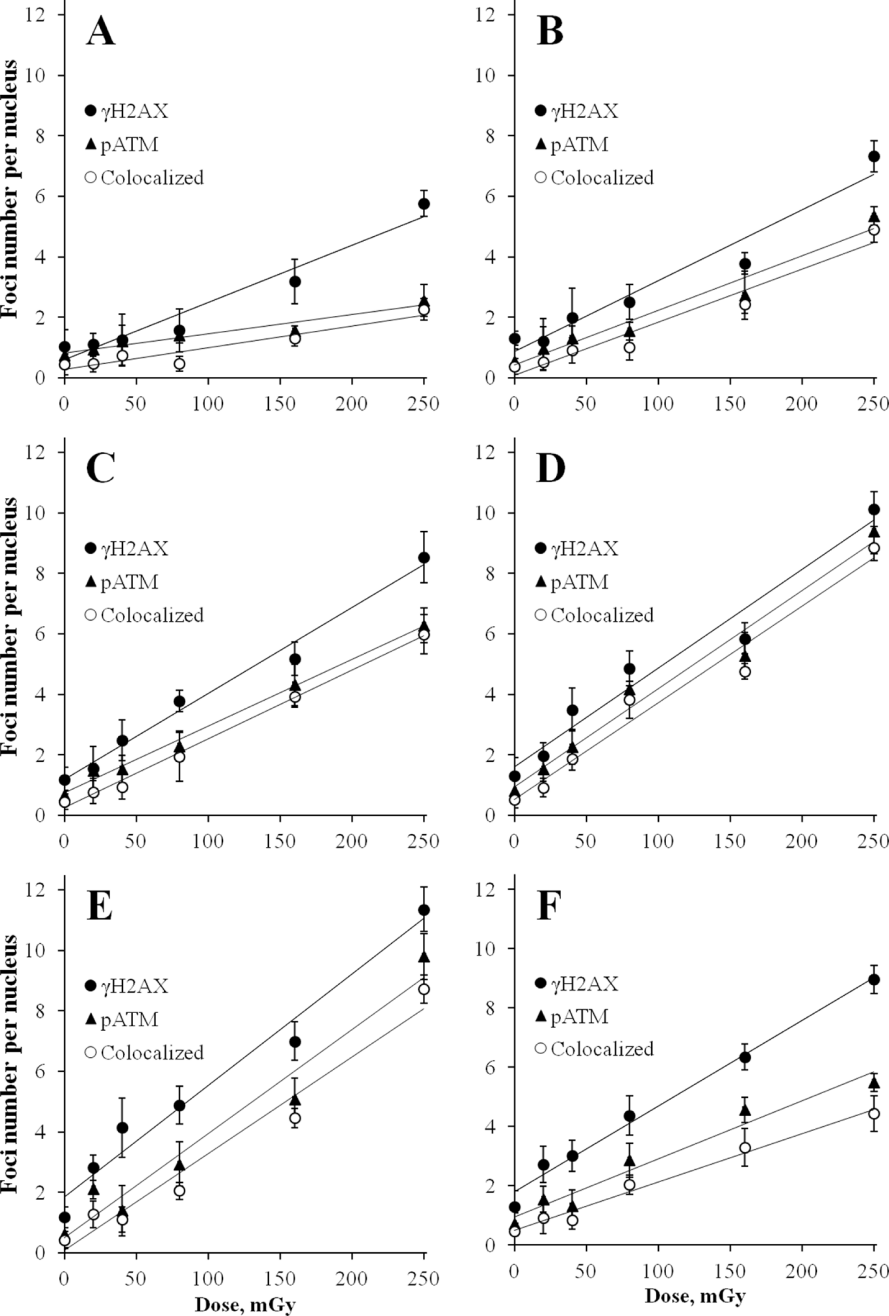
Radiation dose-responses for γH2AX and pATM foci in MSCs Cells were exposed to X-irradiation at various indicated doses and fixed at 5 min **(A)** 10 min **(B)** 15 min **(C)** 30 min **(D)** 60 min **(E)** and 120 min **(F).** Immuonofluorescence labeling for γH2AX and pATM was performed as described in Materials and Methods. Number of foci for each protein and the number of co-localized foci were quantified and mean values of three independent experiments ± SD are shown on the graphs.

**Figure 2:**
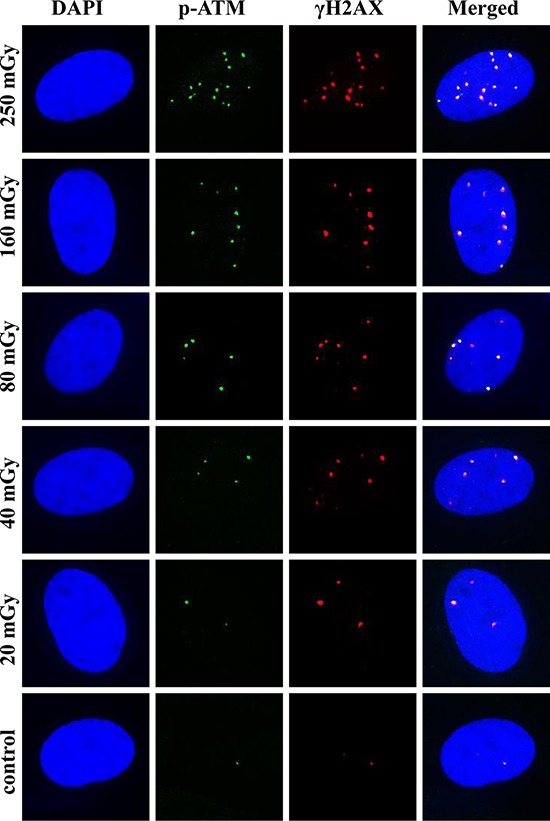
Representative images of γH2AX and pATM foci and their co-localization at 30 min post-irradiation MSCs were irradiated and γH2AX and pATM immunofluorescently labeled as described in Materials and Methods. Representative single cell images are shown for each irradiation dose. Nuclei were counter-stained with DAPI, shown in blue in the first column of images. pATM and γH2AX are shown in green and red, respectively. Images shown in the last column were produced by merging all three channels and show the co-localization pattern of γH2AX and pATM.

Our first set of experiments aimed at detailed characterization of dose-responses for γH2AX and pATM foci in MSCs exposed to X-ray doses of 20, 40, 80, 160 and 250 mGy. Time post-exposure is also an important variable that affects biological response. Phosphorylation of ATM and H2AX occurs very early after exposure and undergoes dynamic changes in time [35, 36, 42]. With this in mind, a broad range of time-points (5, 10, 15, 30, 60 and 120 min) was chosen for dose-response experiments, allowing reconstruction of a detailed pattern of foci formation. Figure 1 shows results of these dose-response experiments for quantification of γH2AX and pATM foci and their co-localization in MSCs. Linear dose-response relationships, represented by straight lines on the graphs, were best fits for all time-points for both γH2AX and pATM. Statistical parameters of linear fits for all time-points for both γH2AX and pATM were as follows: *r* ≥ 0.9, *p* ≤ 0.002, R^2^ ≥ 0.9. Interestingly, as early as 5 min post-irradiation noticeable accumulation of γH2AX foci were found for 160 and 250 mGy (Figure 1A). The average yield of foci per unit of absorbed dose 5 min after exposure was 19 ± 2 and 6 ± 1 foci/cell/Gy for γH2AX and pATM, respectively. The dose-response curves became steeper with time, indicating the expected accumulation of foci with time up to 30–60 min. The maximum responses were observed at 30 or 60 min post-irradiation, followed by a decrease in foci numbers at 120 min. The latter is normally indicative of DNA DSBs rejoining process. Figure 2 illustrates pATM and γH2AX foci appearance in MSCs at 30 min after irradiation with various doses of X-rays. Increases in number of foci of both proteins are clearly seen on the microscopic images in Figure 2.

Foci numbers calculated from the regression equations for γH2AX and pATM were for 15 min 29 ± 2 and 22 ± 1, and for 30 min 33 ± 3 and 32 ± 3 foci/cell/Gy, respectively. At all time-points, numbers of pATM foci were lower than numbers of γH2AX foci. However, the gap between the two types of foci was decreasing with time, reaching the minimum at 30 min, and then increasing again between 30 and 120 min. We also measured co-localization of the two types of foci for all doses and time-points assessed (presented as open circles in Figure 1 and as merged microscopic images in Figure 2). Gamma-H2AX and pATM co-localization patterns, that can reveal functional relationship between the two proteins and its dependence on our experimental conditions, are analyzed and described below. At this point, it can be noted that at all time-points and for all doses used complete co-localization of γH2AX with pATM was a rare event.

### Kinetics of γH2AX and pATM foci numbers

**Figure 3:**
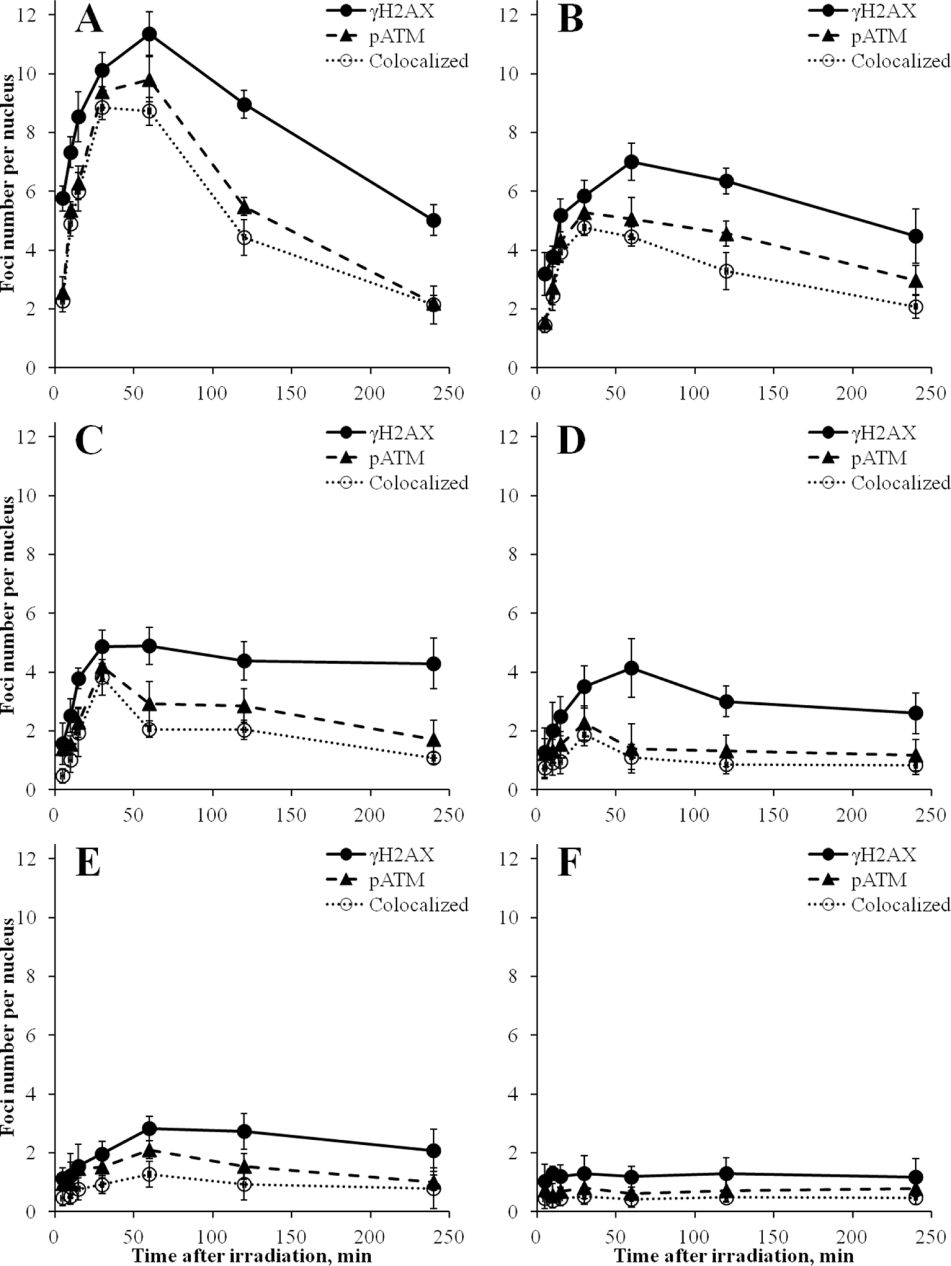
Kinetics of γH2AX, but not pATM, foci induced in MSCs is dose-dependent Cells were exposed to 250 mGy **(A)** 160 mGy **(B)** 80 mGy **(C)** 40 mGy **(D)** 20 mGy **(E)** or left untreated **(F)** and fixed at various indicated time-points after irradiation up to 240 min. Number of γH2AX and pATM foci were quantified, as well as their co-localization and mean values from three independent experiments ± SD were plotted.

Results of measurements of γH2AX and pATM foci numbers in time-course experiments up to 240 min following irradiation of MSCs with various doses of X-rays are presented in Figure 3. Each panel of this Figure shows kinetics of both γH2AX and pATM and their co-localization for a single dose. For the highest dose of 250 mGy, we found the maximum number of γH2AX foci at 60 min post-irradiation, followed by a 60% decrease in the following 3 h (Figure 3A). The number of γH2AX foci at 240 min was significantly lower compared with the number of foci at 60 min (*p* = 0.0004). Similarly, the dose of 160 mGy resulted in the maximum number of γH2AX foci at 60 min (Figure 3B). Subsequently, at 240 min post-irradiation, foci numbers reduced by 40% compared with 60 min time-point (*p* = 0.0487). In contrast, for lower doses of 20, 40 and 80 mGy, no statistically significant differences in γH2AX foci numbers at 240 min compared with 60 min post-irradiation were found, with *p*-values being 0.334, 0.232 and 0.607, respectively (Figures 3C–3F). These results show differential kinetics of γH2AX foci produced by intermediate doses of X-rays (160–250 mGy) compared with low doses (≤ 80 mGy) in MSCs.

**Figure 4:**
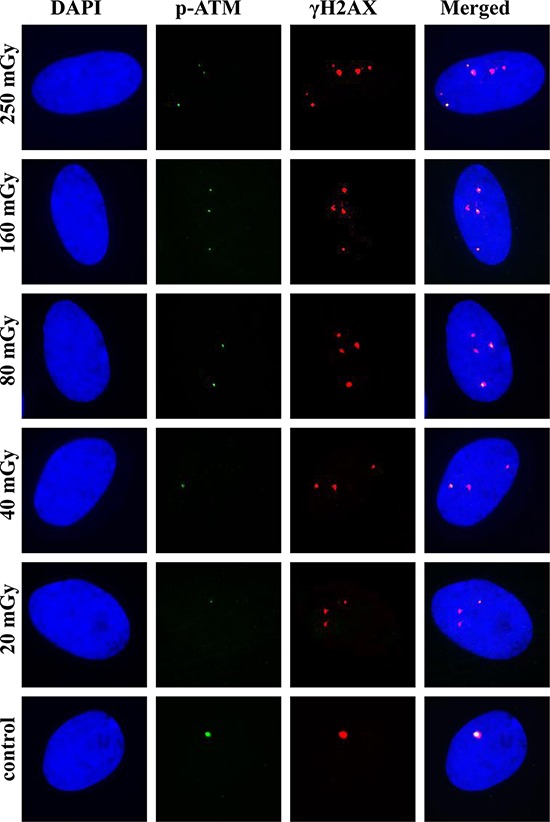
Representative images of γH2AX and pATM foci and their co-localization at 240 min post-irradiation MSCs were irradiated and γH2AX and pATM immunofluorescently labeled as described in Materials and Methods. Representative single cell images are shown for each irradiation dose. Nuclei were counter-stained with DAPI, shown in blue in the first column of images. pATM and γH2AX are shown in green and red, respectively. Images shown in the last column were produced by merging all three channels and show the co-localization pattern of γH2AX and pATM.

For pATM, the maximum accumulation of foci numbers was observed at 30 or 60 min, which was consistent with the data for γH2AX foci (Figure 3). However, their subsequent loss followed a different pattern compared with γH2AX. For all the doses used, pATM foci numbers reduced substantially by 240 min after irradiation, in most cases down to basal levels. In Figure 4, representative images obtained by immunofluorescence microscopy show γH2AX and pATM foci appearance and their co-localization in MSCs at 240 min following exposure to various doses of X-ray radiation. It can be seen that only few radiation-induced pATM foci persisted by 240 min after exposure (compare 1–3 foci in irradiated cells to 1 focus in the control), whereas γH2AX foci in irradiated cells were in excess relative to unirradiated control for all doses, including the lowest 20 mGy dose.

### Differential γH2AX and pATM co-localization patterns for low vs. intermediate doses

**Figure 5:**
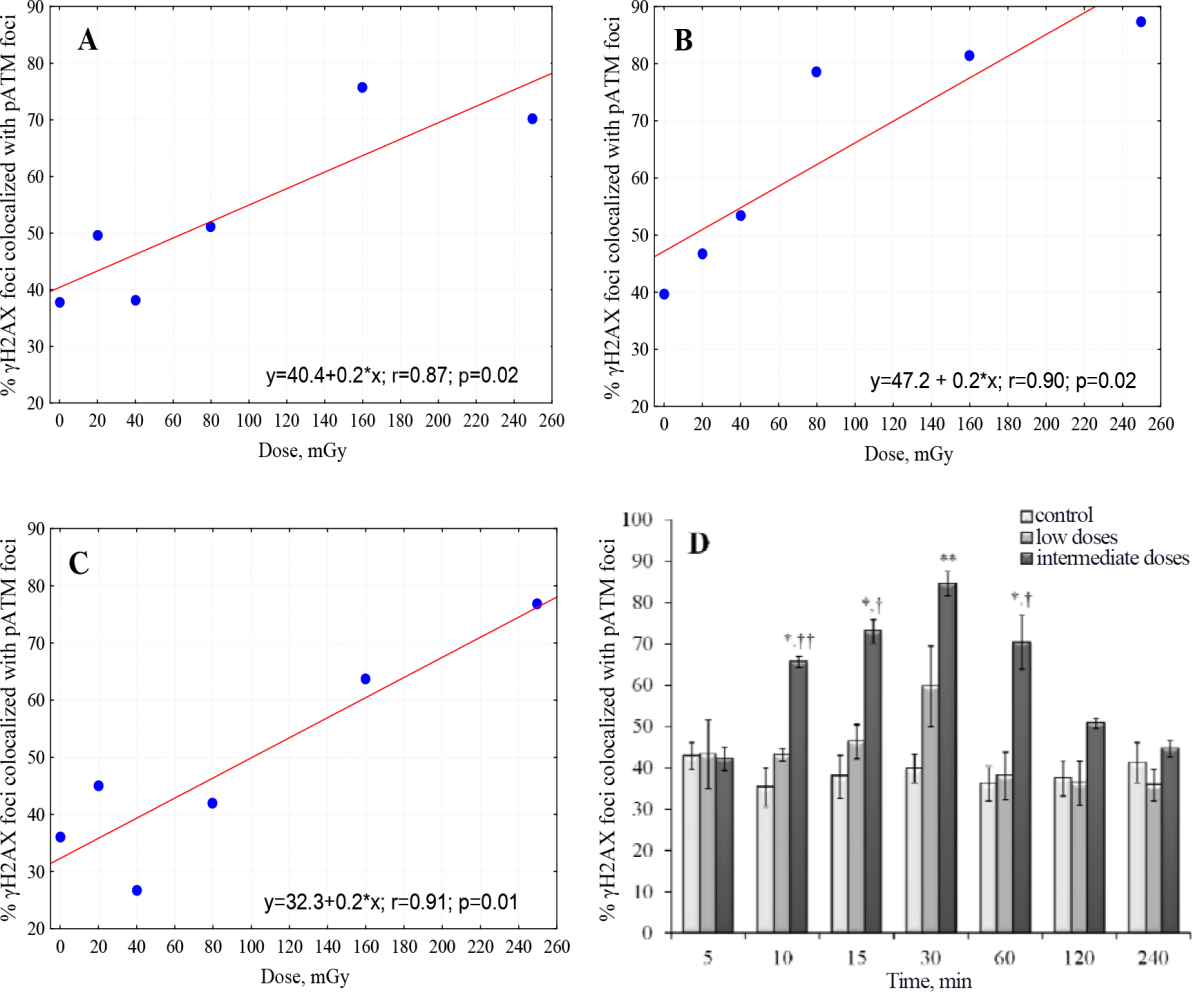
Differential co-localization of γH2AX and pATM foci for low vs. intermediate doses Percent γH2AX foci co-localized with pATM foci was plotted against radiation dose for 15 min **A.** 30 min **B.** and 60 min **C.** time-points post-irradiation. Linear fits used to describe the relationships were statistically significant with the parameters shown on the corresponding plots. **D.** Percent γH2AX foci co-localized with pATM foci were pooled within two separate dose groups of low (20, 40 and 80 mGy) and intermediate (160 and 250 mGy) doses. Resulting cumulative percentages are shown as bars for both dose groups and the control, and for each time-point examined in this study. † and †† denote statistically significant difference between low and intermediate dose groups at *P* < 0.05 and 0.01, respectively. * and ** denote statistically significant difference between control and intermediate dose groups at *P* < 0.05 and 0.01, respectively.

It can be seen from Figures 1 and 3 that, in general, majority of pATM foci were co-localized with γH2AX foci (triangles and open circles are very close to each other). In contrast, the fraction of γH2AX foci co-localized with pATM foci varied greatly (the greater distance between filled and open circles in Figure 1, the poorer co-localization between γH2AX and pATM foci). We noticed that higher co-localization of the two proteins was seen at 30 min after irradiation (almost coinciding closed and open circles in Figure 1D) The greater time distance from the 30 min time-point was, the greater gap between γH2AX and pATM was observed (compare panels D with the rest of panels in Figure 1). This is also evident from comparing merged images in Figures 2 and 4 – only lone γH2AX foci (red foci) were not co-localized with green pATM foci at 30 min for some doses (Figure 2), whereas for every dose a substantial number of non-co-localized foci were observed at 240 min post-irradiation (Figure 4). Furthermore, co-localization pattern appeared to depend on dose. Indeed, poor co-localization was noticeable for lower doses below 160 mGy. To examine the relationship of the degree of γH2AX co-localization with pATM and the dose, we plotted the percentage of co-localization against dose for 15, 30 and 60 min time-points (Figure 5A, 5B and 5C). Correlation analysis revealed statistically significant correlation between the co-localization degree and dose for all three time-points examined (results of statistical analyses are shown in panels A-C of Figure 5). However, within a dose range of 0–80 mGy, only at 30 min, which was the time of maximum foci yields for both γH2AX and pATM, there was a dose-dependent increase in the degree of co-localization (Figure 5B). No such dependence was seen at 15 or 60 min after exposure (Figure 5A and 5C). Therefore, we next grouped doses into a low dose group (20, 40 and 80 mGy) and an intermediate dose group (160 and 250 mGy) and compared the percentage of γH2AX foci co-localized with pATM between the groups for all time-points. Results of such comparison, shown in Figure 5D, revealed significantly poorer co-localization of γH2AX foci with pATM foci for low doses compared with intermediate doses.

## DISCUSSION

Dose-responses provide important basic information for characterization of biological responses to ionizing radiation exposure in a given experimental model. In our study, we used a variety of doses that can be categorized into two groups of low and intermediate doses [43]. The former group consisted of 20, 40 and 80 mGy, representing doses that can be encountered during medical diagnostic or treatment procedures [44], while the latter group included 160 and 250 mGy. Since molecular responses to radiation are dynamic processes which develop and change with time, it is also important to cover multiple time-points, especially for responses related to DNA damage recognition and signaling. Most often, dose-responses for γH2AX foci are studied within 15–60 min post-irradiation [45, 46]. Similar time-points are typically used for measuring foci of pATM that represents the activated form of ATM [47]. Proper activation of ATM is a complex multistage process that normally occurs very early after DNA damage infliction and leads to the formation of γH2AX, via direct phosphorylation of H2AX by pATM [35, 36], and other phosphorylated proteins involved in DNA damage signaling [40, 41], and defines the cell fate. Co-localization of pATM and γH2AX foci is typically regarded as a confirmation of the ATM-dependent nature of γH2AX formation. Our detailed (with respect to time and dose) analyses of γH2AX and pATM foci formation in MSCs revealed several interesting findings described below.

Firstly, we observed linear dose responses for both γH2AX and pATM foci at all time-points used. The maximum response was seen at 60 min for γH2AX and at 30–60 min for pATM, depending on dose. Average foci numbers per cell per Gy calculated mathematically from the linear fits at maximum response times were 37 ± 3 and 32 ± 3 for γH2AX and pATM, respectively. Similar dose responses and foci numbers were reported for other normal human cells within 120 min post-irradiation; however, higher doses were used in that study [48]. This result suggests that early molecular responses to DNA DSBs produced by ionizing radiation in MSCs are normal and similar to those in differentiated cells.

Secondly, and more interestingly, noticeable differences in the kinetics of γH2AX foci compared with pATM foci were observed in the time period between 60 and 240 min post-irradiation (Figure 3). The rate of γH2AX foci loss in MSCs, typically representing the rate of rejoining of DNA DSBs, appears to be dependent on radiation dose: the lower the dose, the lower rejoining rate. Indeed, by 240 min after exposure, 60% of the maximum foci number disappeared for 250 mGy dose; such reduction in foci numbers for 160 mGy was only 40%, and no significant reduction in foci numbers was observed for 20–80 mGy (Figure 3). It is tempting to argue that DNA DSBs produced by low radiation doses are not repaired as efficiently as those produced by intermediate doses. Indeed, long persistence of γH2AX foci was observed for alpha-particle irradiated normal human fibroblasts and was associated with higher complexity of DNA DSBs compared with gamma-radiation induced γH2AX foci that were shown to disappear faster [49]. Similar results were reported for non-proliferating human mammary epithelial cells exposed to iron-ions [50]. However, the doses used in these reports were substantially higher (0.5–2 Gy) than doses used in our study. It is unlikely that the complexity of DNA DSBs would increase with the decrease in dose of low LET radiation, such as X-rays used in this study. Dose-dependent patterns of foci kinetics similar to the ones found in this study were reported for primary cultures of human fibroblasts; however the effects were observed in a lower dose range between 2.3 and 10 mGy [51]. The authors also showed that inducible DNA repair, triggered by pre-exposure to hydrogen peroxide, was required to reduce number of foci after low doses at delayed times post-irradiation, providing another argument against the complexity of DNA DSBs as an explanation for long persistence of γH2AX foci following low doses. Alternative explanation could be *de novo* formation of additional foci that are not necessarily related to radiation-induced DNA DSBs but represent temporary foci occurring in the S-phase at stalled replication forks that can originate from endogenous or induced single-strand breaks [52]. In contrast to γH2AX foci, kinetics of pATM foci did not depend on radiation dose and their efficient removal was observed in the entire dose range studied. Such uncoupled kinetics of foci for the two proteins after exposure to low doses suggests that H2AX phosphorylation status is independent of activated ATM in MSCs.

Thirdly, analysis of γH2AX and pATM co-localization patterns showed that it depends on radiation dose (Figure 5A, 5B and 5C). Even more strikingly, poor correlations were seen between γH2AX and pATM co-localization and dose, when examined separately within low and intermediate dose ranges. It follows then, that the two dose groups are qualitatively different in terms of how molecular responses to DNA DSBs are realized in MSCs. Those differences are seen as i) long persistence of γH2AX foci and ii) poor co-localization of γH2AX and pATM foci - for low, but not intermediate, doses. The latter is clearly demonstrated in Figure 5D, whereby statistically significant differences in γH2AX and pATM co-localization levels are seen between the low and intermediate dose groups (the data were pooled for 20–80 mGy for the low dose group and for 160 and 250 mGy for the intermediate dose group).

Noteworthy, the co-localization levels for low doses were very similar to the ones observed for unirradiated control cells for all time-points except for 30 min, when the response reached its near maximum level. Low co-localization of γH2AX and pATM at early time points may be explained by low number of pATM foci early after exposure. This can be due to detection limit and/or the fact that ATM activation, occurring 10–60 min after infliction of DNA damage, is thought to be preceded by very early (2–10 min) activation of DNA-PKcs that is also a kinase able to phosphorylate H2AX [42]. Besides, activation of ATM is a complex multistep process. First, inactive ATM dimers are dissociated as a result of interaction with the MRN complex activated by DNA DSBs [35, 53]. Such partially activated ATM is able to phosphorylate some proteins, including histone H2AX. However, for full activation of ATM the two-stage autophosphorylation of this kinase is required [54, 55].

As mentioned before, the lack of γH2AX and pATM foci co-localization 60–240 min following irradiation with low doses may point to a non-radiation nature of foci or DNA DSBs. γH2AX foci can be formed in senescent cells in which case they are not directly related to DNA damage or at least to exogenously induced DNA damage [56, 57]. However, stress-induced premature senescence normally requires long periods of time (days to weeks) following stress exposure to develop [58]; therefore, this possibility as an explanation for our results obtained within 4 h following irradiation can be ruled out. Histone H2AX can be phosphorylated not only by the ATM kinase, but also by the DNA-PKcs and ATR kinases, activation of which is not always triggered by radiation-induced DNA DSBs [36, 59]. ATR is activated primarily in S phase in response to collapse of replication forks [60, 61]. In contrast to ATR, the ATM activity in S phase does not change significantly, and spontaneous phosphorylation of ATM is rarely observed [47]. Simultaneous hyper-production of free radicals, which is often observed after exposure to ionizing radiation at low doses [62, 63], and stimulation of MSCs proliferation (e.g. such as that reported for rat bone marrow MSCs [23]), can ultimately lead to replication stress and generation of secondary DNA DSBs. Those DSBs could then be seen as *de novo* γH2AX foci at > 120 min after irradiation and would be pATM-independent. This scenario very closely resembles the patterns of γH2AX and pATM foci observed in our study. Thus, we are inclined to believe that the observed long persistence of γH2AX foci following exposure to low doses is indicative of changes in DNA metabolism, such as DNA replication, rather than of inefficient repair of radiation-induced DNA DSBs in MSCs. Interestingly, DNA DSBs resulting from the collapse of replication forks are repaired by a slow but more accurate mechanism of homologous recombination [64] and therefore generally represent lower hazard with respect to the ultimate cell fate compared with radiation-induced DSBs.

Further investigations are warranted to understand the biological significance of the observed differential γH2AX foci responses to low compared with intermediate doses in MSCs and its apparent independence of pATM for clinical applications. In particular, the nature of the long persistence of γH2AX foci needs to be examined. Are they *de novo* formed metabolic DNA DSBs that are repaired efficiently by homologous recombination or are they unrepaired or misrepaired radiation-induced breaks? Although experimental evidence exists to support both scenarios, the former appears to have stronger support and to be more realistic and logical. Furthermore, certain interest represents a question whether such peculiar molecular responses to low doses can affect functions of the MSCs - can they alter the choice of differentiation between orthodox (mesodermal lineages – adipo-, osteo-, chondrogenic differentiation) and non-orthodox (ectodermal and endodermal lineages – neuro-, myogenic differentiation etc.) directions? Will they interfere with the MSCs interaction with host tissues upon therapeutic medical procedures? Current results provide the basis for further studies in these directions related to the use of MSCs in the clinics.

## MATERIALS AND METHODS

### Human gingival MSCs isolation, cultivation and characterization

Experiments were performed on MSCs cultures isolated from oral mucosa (gingiva) biopsy specimens of 4 healthy volunteers (women 26–32 years old). All donors signed the informed consent before procedure. The biopsy specimens were placed in Dulbecco's Modified Eagles Medium (DMEM; StemCell Technology, USA) supplemented with 5% Fetal Bovine Serum (FBS; Biological Industries, Israel), 1 g/L D-glucose, 200 U/mL penicillin and 200 ug/mL streptomycin, and immediately transported to cell culture laboratory. Tissue samples were minced using sterile disposable scalpels. Homogenates were incubated with 1 mL of 0.25% Trypsin/EDTA (StemCell Technology, USA) at 37°C for 1 hour. Enzyme activity was blocked by adding 1 mL FBS (Biological Industries, Israel). After that homogenates were centrifuged 7 minutes at 300 g. Obtained suspensions were incubated in 1 ml of 0.15% collagenase type II (Sigma, USA) at 37°C for 2 hours. Enzyme activity was blocked by adding 1 mL FBS (Biological Industries, Israel), cell suspensions were centrifuged 7 minutes at 300 g. Pellets were resuspended in culture medium MesenCult (StemCell Technology, USA). Cell count and cell viability assessment were performed using an automatic cell counter Countess (Invitrogen, USA). Cell number isolated from gingiva biopsy specimens was 7.88 × 10^6^ ± 0.4 × 10^6^. Cells were seeded in culture flasks at the density of 3 × 10^5^ cells/cm^2^ and incubated at 37°C and 5% CO_2_. Expansion of cells was performed according to standard procedures of MSCs cultivation. MSCs were cultured up to passage 2 at 75–80% of confluency with medium changed every three days. Differentiation of gingiva derived MSCs into the chondrogenic, adipogenic and osteogenic directions was performed using Human Mesenchymal Stem Cell Functional Identification Kit (R&D Systems, USA) according to the manufacturer's procedure.

For immunophenotypic characterization, cells at passage 2 were detached using 0.05% Trypsin/EDTA (StemCell Technology, USA), washed and counted. Ten to twenty thousand cells in phosphate buffered saline (PBS) were stained with labeled antibodies according to the manufacturer's instructions. Phenotyping was performed on a flow cytometer BD FACS Canto II (USA). For the identification and characterization of the cultured cells, the following set of monoclonal antibodies was used: CD90, CD73, CD105, CD54, CD44, CD13, CD34, CD117, CD45, CD14 (all from BD Bioscience, USA). The antibodies detect typical markers of mesenchymal progenitor cells. FITC-, APC-, PerCp- and PE-labeled IgG antibodies of corresponding class were used as isotype controls. Gingiva derived MSCs had a high level of expression of CD44, CD13, CD90, CD105, CD73, did not express markers of progenitor hematopoietic (CD34, CD45, CD14, CD117) cells, and had a low level of expression of adhesion molecule (CD54).

For irradiation experiments MSCs at passage 2 were detached, washed, resuspended and seeded at the density of 5 × 10^3^ cells/cm^2^ in 500 μL of culture medium onto coverslips (SPL Lifesciences, South Korea) placed inside 35 mm Petri dishes (Corning, USA). To improve adhesion of cells additional volume of culture medium (1, 5 mL) was added into Petri dishes 15 minutes after seeding. Cells seeded on coverslips were incubated at 37°C and 5% CO_2_ for at least 20 h prior to irradiation experiments.

### Irradiation

Cells were exposed to 100 kV X-rays at a dose rate of 40 mGy/min (0.8 mA, 1.5 mm A1 filter) using RUB RUST-M1 X-irradiator (Russia). Throughout the irradiation, cells were maintained at 4°C using a thermo-granules Lab Armour (Life Technologies, USA). The error of exposure dose were calculated to be within 15%. Cells were returned to normal growth conditions immediately after irradiation and maintained for various periods of time before fixation.

### Immunofluorescence microscopy

Cells were fixed on coverslips in 4% paraformaldehyde in PBS (pH 7.4) for 20 min at room temperature followed by two rinses in PBS and permeabilization in 0.3% Triton-X100 (in PBS, pH 7.4) supplemented with 2% bovine serum albumin (BSA) to block non-specific antibody binding. Cells were then incubated for 1 hour at room temperature with primary rabbit monoclonal antibody against γH2AX (clone EP854(2)Y, Merck-Millipore, USA) and primary mouse monoclonal antibody against phosphorylated ATM protein (clone 10H11.E12, Merck-Millipore, USA) which were diluted in PBS (1:200 and 1:400, respectively) with 1% BSA. Following several rinses with PBS, cells were incubated for 1 hour at room temperature with secondary antibodies IgG (H+L) goat anti-mouse (Alexa Fluor 488 conjugated, dilution 1:600; Merck-Millipore, USA) and goat anti-rabbit (rhodamine conjugated, dilution 1:400; Merck-Millipore, USA) diluted in PBS (pH 7.4) with 1% BSA. Coverslips were then rinsed several times with PBS and mounted on microscope slides with ProLong Gold medium (Life Technologies, USA) with DAPI for DNA counter-staining. Cells were viewed and imaged using Nikon Eclipse Ni-U microscope (Nikon, Japan) equipped with a high definition camera ProgRes MFcool (Jenoptik AG, Germany). Filter sets used were UV-2E/C (340–380 nm excitation and 435–485 nm emission) and Y-2E/C (540–580 nm excitation and 600–660 nm emission). At least 200 cells per data point were imaged. Foci were enumerated using Focicounter (http://focicounter.sourceforge.net/).

### Statistical analysis

Statistical and mathematical analyses of the data were conducted using the Statistica 8.0 software (StatSoft). The results are presented as means of three independent experiments ± standard error. Statistical significance was tested using the Student *t*-test.

## References

[1] Cipriani P, Ruscitti P, Di Benedetto P, Carubbi F, Liakouli V, Berardicurti O, Ciccia F, Triolo G, Giacomelli R (2015). Mesenchymal stromal cells and rheumatic diseases: new tools from pathogenesis to regenerative therapies. Cytotherapy.

[2] Sharma RR, Pollock K, Hubel A, McKenna D (2014). Mesenchymal stem or stromal cells: a review of clinical applications and manufacturing practices. Transfusion.

[3] Caplan AI (1991). Mesenchymal stem cells. Journal of orthopaedic research: official publication of the Orthopaedic Research Society.

[4] Bianco P, Robey PG, Simmons PJ (2008). Mesenchymal stem cells: revisiting history, concepts, and assays. Cell stem cell.

[5] Hematti P (2012). Mesenchymal stromal cells and fibroblasts: a case of mistaken identity?. Cytotherapy.

[6] Haniffa MA, Collin MP, Buckley CD, Dazzi F (2009). Mesenchymal stem cells: the fibroblasts’ new clothes?. Haematologica.

[7] Zuk PA, Zhu M, Mizuno H, Huang J, Futrell JW, Katz AJ, Benhaim P, Lorenz HP, Hedrick MH (2001). Multilineage cells from human adipose tissue: implications for cell-based therapies. Tissue engineering.

[8] Oswald J, Boxberger S, Jorgensen B, Feldmann S, Ehninger G, Bornhauser M, Werner C (2004). Mesenchymal stem cells can be differentiated into endothelial cells *in vitro*. Stem cells.

[9] Caplan AI (2000). Mesenchymal stem cells and gene therapy. Clinical orthopaedics and related research.

[10] Robey PG (2011). Cell sources for bone regeneration: the good, the bad, and the ugly (but promising). Tissue engineering Part B, Reviews.

[11] Sekiya I, Larson BL, Smith JR, Pochampally R, Cui JG, Prockop DJ (2002). Expansion of human adult stem cells from bone marrow stroma: conditions that maximize the yields of early progenitors and evaluate their quality. Stem cells.

[12] Zorin VL, Komlev VS, Zorina AI, Khromova NV, Solovieva EV, Fedotov AY, Eremin II, Kopnin PB (2014). Octacalcium phosphate ceramics combined with gingiva-derived stromal cells for engineered functional bone grafts. Biomedical materials.

[13] Sakaguchi Y, Sekiya I, Yagishita K, Muneta T (2005). Comparison of human stem cells derived from various mesenchymal tissues: superiority of synovium as a cell source. Arthritis and rheumatism.

[14] Mitrano TI, Grob MS, Carrion F, Nova-Lamperti E, Luz PA, Fierro FS, Quintero A, Chaparro A, Sanz A (2010). Culture and characterization of mesenchymal stem cells from human gingival tissue. Journal of periodontology.

[15] Fournier BP, Larjava H, Hakkinen L (2013). Gingiva as a source of stem cells with therapeutic potential. Stem cells and development.

[16] Kotenko K, Moroz B, Nadezhina N, Galstyan I, Eremin I, Deshevoy J, Lebedev V, Slobodina T, Grinakovskaya D, Zhgutov Y, Bushmanov A (2012). Successful treatment of localised radiation lesions in rats and humans by mesenchymal stem cell transplantation. Radiation protection dosimetry.

[17] Xu X, Chen C, Akiyama K, Chai Y, Le AD, Wang Z, Shi S (2013). Gingivae contain neural-crest- and mesoderm-derived mesenchymal stem cells. Journal of dental research.

[18] Marynka-Kalmani K, Treves S, Yafee M, Rachima H, Gafni Y, Cohen MA, Pitaru S (2010). The lamina propria of adult human oral mucosa harbors a novel stem cell population. Stem cells.

[19] Treves-Manusevitz S, Hoz L, Rachima H, Montoya G, Tzur E, Vardimon A, Narayanan AS, Amar S, Arzate H, Pitaru S (2013). Stem cells of the lamina propria of human oral mucosa and gingiva develop into mineralized tissues *in vivo*. Journal of clinical periodontology.

[20] Tomar GB, Srivastava RK, Gupta N, Barhanpurkar AP, Pote ST, Jhaveri HM, Mishra GC, Wani MR (2010). Human gingiva-derived mesenchymal stem cells are superior to bone marrow-derived mesenchymal stem cells for cell therapy in regenerative medicine. Biochemical and biophysical research communications.

[21] Huang GT, Gronthos S, Shi S (2009). Mesenchymal stem cells derived from dental tissues vs. those from other sources: their biology and role in regenerative medicine. Journal of dental research.

[22] Alessio N, Del Gaudio S, Capasso S, Di Bernardo G, Cappabianca S, Cipollaro M, Peluso G, Galderisi U (2015). Low dose radiation induced senescence of human mesenchymal stromal cells and impaired the autophagy process. Oncotarget.

[23] Liang X, So YH, Cui J, Ma K, Xu X, Zhao Y, Cai L, Li W (2011). The low-dose ionizing radiation stimulates cell proliferation via activation of the MAPK/ERK pathway in rat cultured mesenchymal stem cells. Journal of radiation research.

[24] Jackson SP, Bartek J (2009). The DNA-damage response in human biology and disease. Nature.

[25] Kotenko KV, Bushmanov AY, Ozerov IV, Guryev DV, Anchishkina NA, Smetanina NM, Arkhangelskaya EY, Vorobyeva NY, Osipov AN (2013). Changes in the number of double-strand DNA breaks in Chinese hamster V79 cells exposed to gamma-radiation with different dose rates. International journal of molecular sciences.

[26] Harper JW, Elledge SJ (2007). The DNA damage response: ten years after. Molecular cell.

[27] Osipov AN, Buleeva G, Arkhangelskaya E, Klokov D (2013). *In vivo* gamma-irradiation low dose threshold for suppression of DNA double strand breaks below the spontaneous level in mouse blood and spleen cells. Mutation research.

[28] Osipov AN, Lizunova EY, Gur'ev DV, Vorob'eva NY (2011). Genome damage and reactive oxygen species production in the progenies of irradiated CHO-K1 cells. Biophysics.

[29] Rodriguez-Rocha H, Garcia-Garcia A, Panayiotidis MI, Franco R (2011). DNA damage and autophagy. Mutation research.

[30] Taleei R, Girard PM, Nikjoo H (2015). DSB repair model for mammalian cells in early S and G1 phases of the cell cycle: Application to damage induced by ionizing radiation of different quality. Mutation research Genetic toxicology and environmental mutagenesis.

[31] Rothkamm K, Lobrich M (2003). Evidence for a lack of DNA double-strand break repair in human cells exposed to very low x-ray doses. Proceedings of the National Academy of Sciences of the United States of America.

[32] Collis SJ, Schwaninger JM, Ntambi AJ, Keller TW, Nelson WG, Dillehay LE, Deweese TL (2004). Evasion of early cellular response mechanisms following low level radiation-induced DNA damage. The Journal of biological chemistry.

[33] Kinner A, Wu W, Staudt C, Iliakis G (2008). Gamma-H2AX in recognition and signaling of DNA double-strand breaks in the context of chromatin. Nucleic acids research.

[34] Paull TT, Rogakou EP, Yamazaki V, Kirchgessner CU, Gellert M, Bonner WM (2000). A critical role for histone H2AX in recruitment of repair factors to nuclear foci after DNA damage. Current biology: CB.

[35] Burma S, Chen BP, Murphy M, Kurimasa A, Chen DJ (2001). ATM phosphorylates histone H2AX in response to DNA double-strand breaks. The Journal of biological chemistry.

[36] Stiff T, O'Driscoll M, Rief N, Iwabuchi K, Lobrich M, Jeggo PA (2004). ATM and DNA-PK function redundantly to phosphorylate H2AX after exposure to ionizing radiation. Cancer research.

[37] Zhou BB, Elledge SJ (2000). The DNA damage response: putting checkpoints in perspective. Nature.

[38] O'Driscoll M, Ruiz-Perez VL, Woods CG, Jeggo PA, Goodship JA (2003). A splicing mutation affecting expression of ataxia-telangiectasia and Rad3-related protein (ATR) results in Seckel syndrome. Nature genetics.

[39] Reitsema T, Klokov D, Banath JP, Olive PL (2005). DNA-PK is responsible for enhanced phosphorylation of histone H2AX under hypertonic conditions. DNA repair (Amst).

[40] Lee JH, Paull TT (2007). Activation and regulation of ATM kinase activity in response to DNA double-strand breaks. Oncogene.

[41] Kurz EU, Less-Miller SP (2004). DNA damage-induced activation of ATM and ATM-dependent signaling pathways. DNA repair.

[42] Flassig RJ, Maubach G, Tager C, Sundmacher K, Naumann M (2014). Experimental design, validation and computational modeling uncover DNA damage sensing by DNA-PK and ATM. Molecular biosystems.

[43] United Nations (2015). Scientific Committee on the Effects of Atomic Radiation. Sources and effects of ionizing radiation: United Nations Scientific Committee on the Effects of Atomic Radiation: UNSCEAR 2012 report to the General Assembly, with scientific annexes.

[44] Nguyen PK, Wu JC (2011). Radiation exposure from imaging tests: is there an increased cancer risk?. Expert review of cardiovascular therapy.

[45] Lobrich M, Shibata A, Beucher A, Fisher A, Ensminger M, Goodarzi AA, Barton O, Jeggo PA (2010). gammaH2AX foci analysis for monitoring DNA double-strand break repair: strengths, limitations and optimization. Cell cycle.

[46] Neumaier T, Swenson J, Pham C, Polyzos A, Lo AT, Yang P, Dyball J, Asaithamby A, Chen DJ, Bissell MJ, Thalhammer S, Costes SV (2012). Evidence for formation of DNA repair centers and dose-response nonlinearity in human cells. Proceedings of the National Academy of Sciences of the United States of America.

[47] Suzuki K, Okada H, Yamauchi M, Oka Y, Kodama S, Watanabe M (2006). Qualitative and quantitative analysis of phosphorylated ATM foci induced by low-dose ionizing radiation. Radiation research.

[48] Mariotti LG, Pirovano G, Savage KI, Ghita M, Ottolenghi A, Prise KM, Schettino G (2013). Use of the gamma-H2AX assay to investigate DNA repair dynamics following multiple radiation exposures. PloS one.

[49] Antonelli F, Campa A, Esposito G, Giardullo P, Belli M, Dini V, Meschini S, Simone G, Sorrentino E, Gerardi S, Cirrone GA, Tabocchini MA (2015). Induction and Repair of DNA DSB as Revealed by H2AX Phosphorylation Foci in Human Fibroblasts Exposed to Low- and High-LET Radiation: Relationship with Early and Delayed Reproductive Cell Death. Radiation research.

[50] Groesser T, Chang H, Fontenay G, Chen J, Costes SV, Helen Barcellos-Hoff M, Parvin B, Rydberg B (2011). Persistence of gamma-H2AX and 53BP1 foci in proliferating and non-proliferating human mammary epithelial cells after exposure to gamma-rays or iron ions. International journal of radiation biology.

[51] Grudzenski S, Raths A, Conrad S, Rube CE, Lobrich M (2010). Inducible response required for repair of low-dose radiation damage in human fibroblasts. Proceedings of the National Academy of Sciences of the United States of America.

[52] Saleh-Gohari N, Bryant HE, Schultz N, Parker KM, Cassel TN, Helleday T (2005). Spontaneous homologous recombination is induced by collapsed replication forks that are caused by endogenous DNA single-strand breaks. Molecular and cellular biology.

[53] Kobayashi J (2004). Molecular mechanism of the recruitment of NBS1/hMRE11/hRAD50 complex to DNA double-strand breaks: NBS1 binds to gamma-H2AX through FHA/BRCT domain. Journal of radiation research.

[54] Bakkenist CJ, Kastan MB (2003). DNA damage activates ATM through intermolecular autophosphorylation and dimer dissociation. Nature.

[55] Dupre A, Boyer-Chatenet L, Gautier J (2006). Two-step activation of ATM by DNA and the Mre11-Rad50-Nbs1 complex. Nature structural & molecular biology.

[56] Pospelova TV, Demidenko ZN, Bukreeva EI, Pospelov VA, Gudkov AV, Blagosklonny MV (2009). Pseudo-DNA damage response in senescent cells. Cell cycle.

[57] Darzynkiewicz Z (2009). When senescence masquerades as DNA damage: is DNA replication stress the culprit?. Cell cycle.

[58] Toussaint O, Medrano EE, von Zglinicki T (2000). Cellular and molecular mechanisms of stress-induced premature senescence (SIPS) of human diploid fibroblasts and melanocytes. Experimental gerontology.

[59] Ward IM, Chen J (2001). Histone H2AX is phosphorylated in an ATR-dependent manner in response to replicational stress. The Journal of biological chemistry.

[60] Cimprich KA, Cortez D (2008). ATR: an essential regulator of genome integrity. Nature reviews Molecular cell biology.

[61] Ward IM, Minn K, Chen J (2004). UV-induced ataxia-telangiectasia-mutated and Rad3-related (ATR) activation requires replication stress. The Journal of biological chemistry.

[62] Large M, Reichert S, Hehlgans S, Fournier C, Rodel C, Rodel F (2014). A non-linear detection of phospho-histone H2AX in EA. hy926 endothelial cells following low-dose X-irradiation is modulated by reactive oxygen species. Radiation oncology.

[63] Baulch JE, Craver BM, Tran KK, Yu L, Chmielewski N, Allen BD, Limoli CL (2015). Persistent oxidative stress in human neural stem cells exposed to low fluences of charged particles. Redox biology.

[64] Petermann E, Helleday T (2010). Pathways of mammalian replication fork restart. Nature reviews Molecular cell biology.

